# Reply to Comment on “Combination of the Fibrosis-4 Index and Carbohydrate Antigen 125 to Predict Morbidity and Mortality in Acute Heart Failure”

**DOI:** 10.31083/RCM51120

**Published:** 2026-04-22

**Authors:** Franco Appiani, Raquel López-Vilella, Luis Almenar-Bonet

**Affiliations:** ^1^Heart Failure and Transplant Unit, Hospital Universitari i Politècnic La Fe, 46007 Valencia, Spain; ^2^Cardiology Department, Hospital Universitari i Politècnic La Fe, 46007 Valencia, Spain; ^3^Centro de Investigación Biomédica en Red de Enfermedades Cardiovasculares (CIBERCV), Instituto de Salud Carlos III, 28029 Madrid, Spain

Dear Editor,

We thank the authors for their thoughtful and constructive commentary on our study evaluating the combined use of the fibrosis-4 index (FIB-4) and carbohydrate antigen 125 (CA125) in patients hospitalized with acute heart failure (AHF) [1]. We appreciate their collegial tone and their emphasis on pragmatic, widely available biomarkers to inform risk stratification in a clinically heterogeneous syndrome.

As highlighted in our report, the study was designed with a particular focus on morbidity and clinical instability. In a cohort of 402 consecutive AHF admissions, patients with concomitantly elevated FIB-4 (≥1.3) and CA125 (>50 U/mL) exhibited a significantly higher incidence of worsening heart failure episodes, whereas survival differences were not observed within the follow-up period.

We agree with the reviewers that fibrosis-based indices may provide multidimensional prognostic information, potentially complementing congestion biomarkers such as CA125. This perspective is consistent with the biological premise that, although both markers relate to congestion, CA125 predominantly reflects serosal and interstitial fluid dynamics and mesothelial activation, while FIB-4 integrates additional domains, including hepatic involvement and systemic inflammatory burden [2, 3]. The authors’ contribution regarding the fibrosis-5 index (FIB-5) is particularly relevant, as its calculation incorporates albumin and alkaline phosphatase in addition to variables shared with FIB-4 [4]. In the setting of AHF, these additional parameters may provide complementary information beyond hepatocellular injury alone, as serum albumin has consistently been associated with systemic vulnerability and adverse prognosis in heart failure, while alkaline phosphatase may reflect cholestatic or congestive hepatic involvement within the cardio-hepatic interaction [5, 6]. Thus, FIB-4 and FIB-5 may be viewed not as strictly competing markers, but rather as partially overlapping tools that emphasize different biological dimensions of risk, including hepatic injury, synthetic reserve, venous congestion, and overall systemic frailty.

At the same time, we acknowledge an important limitation regarding direct comparison between the two indices in our own cohort. Although validating the predictive performance of FIB-5 in our dataset would have been highly informative, this was not feasible because the prospective data collection of the original study did not include a systematic assessment of alkaline phosphatase and albumin, which are required for the calculation of FIB-5. Therefore, any post hoc attempt to evaluate FIB-5 would have been incomplete and methodologically unreliable. Future studies specifically designed to collect all components of both indices will be needed to allow a robust head-to-head comparison in patients with AHF.

With respect to timing and reassessment, the reviewer appropriately frames fibrosis-related indices as markers of chronic organ involvement and suggests they may be most informative when assessed at admission or early during hospitalization, whereas CA125 may be reassessed during hospitalization, particularly at discharge, and in early post-discharge follow-up. We concur with this conceptual framework; however, we would like to emphasize that FIB-4 may also have important short-term information when reassessed over relatively brief intervals, including prior to discharge. Available data suggest that serial reassessment of FIB-4 during AHF hospitalization may provide clinically relevant information. In particular, a greater in-hospital reduction in FIB-4 from admission to discharge has been associated with better 180-day outcomes [7, 8]. These findings support the interpretation that, while FIB-4 is commonly viewed as a surrogate of chronic hepatic fibrosis and systemic vulnerability, its short-term trajectory during hospitalization may also capture clinically meaningful hemodynamic improvement and decongestive changes.

Finally, we fully agree with the reviewers regarding confounding conditions and the need for prospective validation. Pre-existing hepatic disease may complicate the interpretation of baseline FIB-related indices, since some of their components may reflect underlying liver disease in addition to cardio-hepatic congestion. However, this does not necessarily invalidate their clinical usefulness. Importantly, most prognostic studies of FIB-4 or FIB-5 in heart failure were performed in broader heart failure cohorts, and some explicitly excluded chronic liver disease, so firm conclusions in patients with cirrhosis or overt hepatic insufficiency are still lacking [6, 9]. Nonetheless, potential confounding of the baseline value does not imply that serial changes during treatment for AHF are not prognostically meaningful. A similar principle applies to N-terminal pro–B-type natriuretic peptide (NT-proBNP): although baseline levels may be influenced by several cardiac and non-cardiac conditions, its percent reduction in-hospital stay remains clinically informative, and a decline of less than 30% has been associated with poorer outcomes [10]. Thus, hepatic disease should be considered a potential confounder that may reduce specificity, rather than a reason to dismiss FIB-related indices altogether.

We thank the authors for their insightful contribution, which enriches the interpretation of multimarker strategies integrating fibrosis-related indices and congestion biomarkers in AHF. We share their view that larger prospective cohorts are needed to define validated thresholds and to develop biomarker-guided care pathways.
